# Supporting Basic Psychological Needs in Medical Education: A Patient Best Practice

**DOI:** 10.5334/pme.1928

**Published:** 2026-03-31

**Authors:** Adam Neufeld, Gregory Guldner

**Affiliations:** 1 Department of Family Medicine, Cumming School of Medicine, University of Calgary, CA; 2 University of California Riverside School of Medicine, US

## Abstract

Physician burnout remains a pervasive challenge in medical education, with significant implications for both physician well-being and the quality and safety of patient care. Despite growing awareness and interventions, medical educators often lack a cohesive theoretical framework that explains how learning environments can contribute to both burnout and clinical performance. This article highlights Self-Determination Theory (SDT) as a robust, evidence-based lens through which to understand and address these challenges by emphasizing the fulfillment of three basic psychological needs: autonomy, competence, and relatedness. Drawing on foundational and contemporary research across health professions education and healthcare, the authors argue that supporting these needs within clinical learning environments not only enhances learner motivation, engagement, and resilience, but also reduces burnout and its downstream effects on empathy, decision-making, teamwork, and patient outcomes. The authors recommend integrating SDT principles into the structure of medical training through curricular design, assessment strategies, faculty development, and institutional policies that support high-quality motivation. They propose that psychological need support should be regarded as a patient best practice and position SDT not only as a wellness framework, but as a clinical quality framework for strengthening learner development, reducing harm, and promoting safe, compassionate, and effective care. Ongoing research, collaboration, and faculty development will be essential to advancing this work across medical education systems.

## 1. Introduction: Burnout, Motivation, and Patient Care

Physician burnout remains a pressing challenge in medical education, with profound consequences for both physician well-being and the quality and safety of patient care [1,2]. While growing awareness has spurred calls for intervention, many solutions remain fragmented, lacking a unifying theoretical foundation to guide sustainable, systemic change. Self-Determination Theory (SDT) [3,4] offers such a foundation. By identifying autonomy, competence, and relatedness as essential psychological needs for motivation and well-being, SDT allows educators and institutions to move beyond generic calls for resilience or self-care. SDT provides a rigorous framework for understanding how the design of the learning environment influences not only learner outcomes but also the quality of care those learners go on to provide. More broadly, SDT has been applied extensively in healthcare contexts, where autonomy-supportive, need-fulfilling environments reliably predict better motivation, mental health, and health behaviours among patients [5].

Prior work has explored how SDT enhances learner engagement, academic performance, and well-being across medical training [6,7,8]. Yet, the field has not fully articulated how disruptions to autonomy, competence, and relatedness impair the very cognitive, emotional, and relational capacities that underlie safe, high-quality patient care. What remains underexamined, and what distinguishes this paper from previous publications on SDT in medical education, is how psychological need support within clinical learning environments translates into downstream effects on patient-centeredness, quality of care, and safety. This paper addresses that gap by foregrounding the motivational mechanisms through which training environments influence clinical performance, advancing the argument that psychological need support constitutes a patient care imperative. In doing so, we position psychological need support not simply as a wellness initiative, but as a scientifically grounded best practice for optimizing patient care—one which strengthens both trainee development and the core mission of medicine: delivering safe, compassionate, and effective care.

We use the term “best practice” in its evidence-based sense: an approach supported by strong theoretical and empirical foundations that advance both education and clinical outcomes. As clinician-educators working across community practice and large health systems, we have repeatedly observed that when training environments become more autonomy-supportive—through small changes in supervision, assessment, and scheduling—learners not only report better well-being, but also show greater clinical presence, follow-through with patients, and willingness to engage with complex care. These observations echo SDT’s predictions and the emerging empirical literature and suggest that the current paucity of direct patient-outcome studies reflects a measurement gap rather than a true absence of effect.

## 2. Self-Determination Theory and Its Relevance to Medical Training

SDT posits that human motivation and well-being depend on the fulfillment of three basic psychological needs: autonomy, competence, and relatedness [3,4]. Autonomy refers to the experience of having meaningful choice and rationale in one’s work. This differs from independence—a central developmental goal of medical training—in that autonomy concerns the quality of regulation, not the amount of supervision. Even within highly structured or hierarchical clinical contexts, autonomy support fosters internalization, engagement, and self-determination [9]. Competence involves experiencing effectiveness and growth in one’s skills, while relatedness reflects feeling valued, respected, and connected with colleagues, mentors, and patients. These needs are universal and invariant across cultures, specialties, and stages of training [10,11].

SDT has one of the most extensive evidence bases in psychological science, demonstrating relevance across education, healthcare, and organizational behaviour [4,12]. In medical education, specifically, SDT has been used to explain learning behaviour, professional identity formation, engagement with assessment systems, and trainee well-being [13,14,15,16]. However, most of this work has focused on learner experience and performance rather than explicitly tracing how psychological need support in training environments shapes the care those learners ultimately provide. Illuminating this linkage—from motivational climate to clinical outcomes—is essential for guiding meaningful educational reform.

## 3. Why Support for Psychological Needs Matters for Clinical Training

### 3.1 How Need Support Shapes Trainee Functioning

Foundational empirical work first demonstrated the long-term clinical relevance of autonomy-supportive learning environments. In a landmark longitudinal study, Williams and colleagues (1996) showed that medical students with autonomy-supportive instructors became more autonomously motivated and confident in their patient care. Crucially, these motivational advantages translated into lasting behavioural outcomes: 30 months later, the same trainees received higher patient-rated clinical care scores, providing early evidence that autonomy support in training environments can meaningfully influence the quality of care delivered.

Complementing this foundational work, Williams and colleagues further demonstrated that autonomy-supportive clerkship environments also shape enduring professional trajectories [17]. In their multi-site study of fourth year students, perceived autonomy support predicted the likelihood of entering internal medicine or surgery, even after controlling for pre-existing career preferences. This effect was mediated by competence and interest, illustrating classic SDT mechanisms and showing that autonomy-supportive learning climates shape not only immediate motivation but also the specialties, and thus the types of clinical work, that physicians ultimately pursue. Although specialty choice is not itself a patient outcome, alignment between personal values and professional pathways fosters engagement, purpose, and sustained quality in clinical practice.

A substantial body of research further demonstrates that when trainees experience their environments as need-supportive, they show higher engagement, deeper learning, better academic performance, and greater well-being [7,18,19]. Conversely, need-thwarting conditions such as excessive control, exclusion, and harsh or ambiguous feedback contribute to emotional exhaustion, disengagement, stress, and burnout [15,20,21].

Need frustration narrows attentional bandwidth, undermines empathy, and shifts learners toward protective, performative behaviours that impede learning and clinical presence [22,23]. Chronic psychological insecurity disrupts working memory, reflective capacity, and emotional regulation—core cognitive processes required for diagnostic accuracy, safe decision-making, and compassionate communication [24,25,26]. Taken together, these foundational and contemporary findings illustrate that supporting basic psychological needs enhances not only motivation, but also the cognitive and interpersonal capacities through which clinicians deliver high-quality care [5].

Recent empirical work has also begun linking motivational climate to clinically relevant interpersonal capacities. In a study of medical students, autonomy-supportive learning environments predicted higher need satisfaction and lower need frustration, which in turn were associated with greater clinical empathy [23]. Although this measure captured self-reported rather than patient-reported empathy, the findings illuminate a plausible pathway through which motivationally healthy learning environments cultivate attuned, empathic clinical practice.

### 3.2 How Need Support Influences Clinical Behaviours and Patient Outcomes

Need-supportive environments also foster stronger teamwork, clearer communication, and more effective collaboration [27]—conditions that consistently predict patient satisfaction, adherence, and safety [28,29,30,31]. Meta-analytic evidence further demonstrates that SDT-informed interventions reliably improve motivation, psychological well-being, and health behaviours across diverse clinical contexts, supporting the practicality and scalability of designing interventions that target psychological needs [32]. In sum, converging evidence across eras and methodologies affirms that the motivational climate of training environments exerts enduring influence on clinical reasoning, communication, collaboration, and care quality.

## 4. Burnout As a Threat to Patient Care

Burnout—recognized as a systemic issue rather than an individual failing—has been consistently linked to increased medical errors, safety incidents, impaired decision-making, and reduced empathy [24,33]. Emotional exhaustion and depersonalization reduce cognitive functioning and communication quality, undermining patient trust, satisfaction, and adherence [34,35]. The same motivational logic clarifies why burnout so reliably predicts errors: burnout reflects the chronic frustration of the psychological needs required for optimal functioning [36,37,38].

The drivers of burnout include excessive workloads, persistent structural job demands that impede physicians’ abilities to meet patient and educational goals, toxic workplace cultures, and inadequate institutional support [39,40]. Early career attrition, absenteeism, and impaired teamwork further disrupt continuity of care [41,35].

Recent scholarship has also highlighted micromanagement as a particularly harmful supervisory pattern. A scoping review found that excessive control, undue scrutiny, and low psychological safety consistently undermined autonomy, strained supervisory relationships, hindered professional development, and in some cases, compromised patient care [42]. This research shows how autonomy-thwarting dynamics translate motivational deficits into clinical vulnerability.

Complementary evidence from outside medical education shows that when healthcare professionals’ basic psychological needs are better satisfied, they experience greater work well-being and engagement, even in technologically complex environments [43]. These findings underscore that psychological need satisfaction is a central determinant of clinicians’ functioning, with consequences that extend to patient care.

Although associations between burnout and patient outcomes are well-established, the psychological mechanisms connecting work environments to clinical performance remain underarticulated. SDT provides this missing explanatory lens by demonstrating that unmet psychological needs give rise to the emotional and cognitive vulnerabilities that manifest as burnout, disengagement, and compromised patient care [44,45,46,47,37].

### 4.1 Putting SDT Into Practice in Medical Education

Applying SDT in medical education does not require wholesale system redesign. Rather, it requires intentional alignment of educational practices with the conditions known to support high-quality motivation. Faculty development is essential: autonomy is supported by acknowledging learner perspectives, offering structured choice, and providing rationales for expectations and feedback. Competence is strengthened through well-scaffolded supervision, actionable feedback, and psychologically safe opportunities for practice and reflection. Relatedness is cultivated through mentorship, inclusive team cultures, and relational transparency.

Institutions can operationalize these principles through policies, communication practices, evaluation structures, and learning environment monitoring. Many interventions are low-cost but high-yield and benefit both learner development and patient care. A growing body of SDT-informed scholarship offers practical guidance for translating these principles into everyday clinical teaching. Orsini and colleagues’ review [48] synthesizes empirical evidence on how autonomy, competence, and relatedness can be supported in health professions education, identifying concrete educator actions that reliably promote internalization and deeper learning. Complementing this work, SDT-informed tools in medical education outline specific behaviours that support basic psychological needs across teaching, supervision, assessment, remediation, and policy design [49]. These models emphasize intentional, relational approaches to feedback, scaffolding, and coaching that align with the psychological conditions necessary for high-quality motivation and professional growth. In this way, SDT offers not only a conceptual language but a practical architecture for redesigning clinical learning environments in ways that measurably enhance both training and patient outcomes.

Together, these approaches offer domain-specific, evidence-based strategies for creating motivationally healthy learning climates. They parallel SDT-consistent behaviour change techniques identified in broader learning and health contexts [50], while adapting them to the relational, developmental, and supervisory realities of clinical supervision. Even small shifts, such as acknowledging learner perspectives, structuring choice, and clarifying rationales, can meaningfully enhance autonomy, competence, and relatedness in daily training interactions.

SDT also aligns naturally with diversity, equity, and inclusion efforts. Psychological safety—an essential expression of relatedness—supports belonging for learners of all backgrounds [51]. These inclusive, relationally supportive environments not only strengthen learner development but directly enhance communication quality, cultural humility, and teamwork—factors consistently linked to patient experience and safety [52,53]. Because psychological need support is inherently relational and contextual, it reinforces the goals of equity-oriented education and the creation of just, culturally safe learning environments [54].

### 4.2 Future Directions

Future directions should build on this conceptual synthesis by directly examining how specific SDT-informed educational interventions influence observed clinical behaviours, patient experience metrics, and safety outcomes. Despite strong evidence that psychological need frustration predicts distress and burnout, and that burnout in turn predicts errors, impaired decision-making, and poorer patient outcomes, almost no studies have tested this full pathway—from need support to burnout to patient care—within a single analytic model. Multi-site longitudinal studies that integrate learning environment assessments, burnout trajectories, and patient-reported outcomes would provide the empirical foundation needed to establish psychological need support as a core quality standard in medical education. In parallel, the field would benefit from rigorous testing of faculty development models designed to enhance autonomy-supportive teaching and supervision, and from system-level evaluations of how structural reforms, such as workload redistribution, scheduling policies, and documentation redesign, affect psychological need satisfaction and downstream clinical performance.

## 5. Conclusion: Psychological Needs as a Patient Care Imperative

Supporting basic psychological needs is not solely a strategy for improving learner wellness; it is a foundational practice for safeguarding patient care. SDT offers a coherent, evidence-based explanation for how training environments shape the emotional, cognitive, and relational capacities that define clinical excellence. As the evidence makes clear, need-supportive environments benefit not only the learner but the patient, the team, and the healthcare system. By integrating need-supportive principles into faculty development, assessment, and institutional policy, medical education can move beyond fragmented wellness efforts toward systemic, evidence-based strategies that strengthen learning, reduce harm, and elevate the quality and safety of care.
